# Progressive Dysphagia in Joubert Syndrome: A Report of a Rare Case

**DOI:** 10.7759/cureus.66648

**Published:** 2024-08-11

**Authors:** Courteney Castellano, Jomaries O Gomez Rosado, Alexandra Witt, Rebecca Simon, Dyadin Esharif

**Affiliations:** 1 Dr. Kiran C. Patel College of Osteopathic Medicine, Nova Southeastern University, Fort Lauderdale, USA; 2 Department of Pediatrics, Broward Health Medical Center, Fort Lauderdale, USA; 3 Department of Pediatric Gastroenterology, Broward Health Medical Center, Fort Lauderdale, USA

**Keywords:** feeding difficulty, joubert syndrome and related diseases, neonatal hypotonia, progressive dysphagia, molar tooth sign, joubert syndrome (js)

## Abstract

Joubert syndrome is an uncommon, autosomal recessive disorder characterized by abnormal brain development involving the underdevelopment or absence of the cerebellar vermis. The classic clinical features include developmental delays, hypotonia, abnormal eye movements, and hyperpnea. On brain magnetic resonance imaging (MRI), an essential finding for the diagnosis of Joubert syndrome is a cerebellar and brainstem malformation called the molar tooth sign, characterized by a hypoplastic cerebellar vermis with dysplasia of the superior cerebellar peduncles. Here, we describe a case of a two-month-old female with an atypical presentation of Joubert syndrome. Her initial clinical presentation included respiratory distress and concerns for reflux complicated with aspiration pneumonia. Early recognition of clinical and radiologic findings for Joubert syndrome enables an early diagnosis, and therefore timely interventions for improving the child’s development and quality of life.

## Introduction

Joubert syndrome (JS) is a rare autosomal recessive disorder identified by a distinct mid-hindbrain malformation, hypotonia in infancy, and developmental delay [1]. Upon literature review, its incidence is thought to be 1/100,000 live births, although not precisely determined [2]. Clinical features appear in the newborn period, but clinical diagnosis is often delayed to 33 months on average due to various phenotypes and clinical manifestations [3]. Approximately 34 genes have been identified in the diagnosis of JS, 33 autosomal recessive and one X-linked [4]. Multiple genes have been identified such as the TMEM67, AHI1, CEP 290, and ARL13B. These mutations disrupt the formation and maintenance of primary cilia, leading to impaired development and function of the cerebellum and brainstem. Additionally, defects in other genes have also been implicated, highlighting the complex genetic landscape of the syndrome [1].

JS is a clinical diagnosis with findings including, but not limited to, characteristic facial appearance (prominent forehead, epicanthal folds, upturned nose with evident nostrils, an open mouth, and low-set ears), oculomotor abnormalities, hepatic fibrosis, duodenal atresia, endocrine abnormalities, and abnormal respiratory patterns. Brain MRI shows the characteristic findings of a hypoplastic cerebellar vermis, deepened interpeduncular fossa, and thick elongated cerebellar peduncles, known as the molar tooth sign (MTS), which would confirm the JS diagnosis [5]. We report a two-month-old female infant of Haitian descent with an atypical presentation of JS, initially presenting with hypotonia, choking episodes, and milk regurgitation with feeds.

## Case presentation

**Figure 1:**
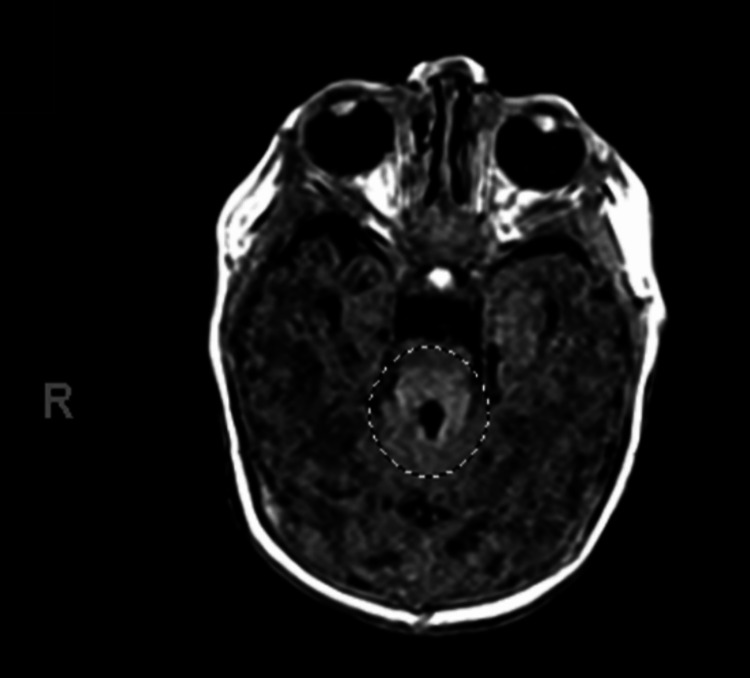
MRI of the brain showing relative hypoplasia of portions of the midbrain with a molar tooth sign as indicated by the circle

We present a two-month-old Haitian-Creole female with a past medical history of Joubert syndrome, patent foramen ovale, ankyloglossia, gastroesophageal reflux disease (GERD), and difficulty feeding who presented to the emergency department due to respiratory distress associated with increased frequency of choking episodes, persistent regurgitation of milk through the nostrils after every feed, and a lethargic appearance. The patient was born at 33 weeks via an emergency C-section due to non-reassuring heart rate tracing, polyhydramnios, and breech presentation. At birth, she had poor tone and respiratory effort requiring non-invasive oxygen supplementation for five days. Additionally, she was found to have abnormal facial features, including a broad forehead, arched eyebrows, open mouth triangular configuration, and hypertelorism. She also presented with 3 anterior tongue cysts, episodes of apnea and hyperpnea, coloboma of the retina, and feeding difficulties. An initial video swallow study completed at three weeks of age showed mild oropharyngeal dysphagia and no aspiration. She required nasogastric feeds and eventually transitioned to oral feeds. MRI of the brain showed the molar tooth sign (Figure 1). Since the child exhibited hypotonia and developmental delays, including feeding difficulties, the presence of the molar tooth sign confirmed the diagnosis. Genetic analysis was not available.

**Figure 2:**
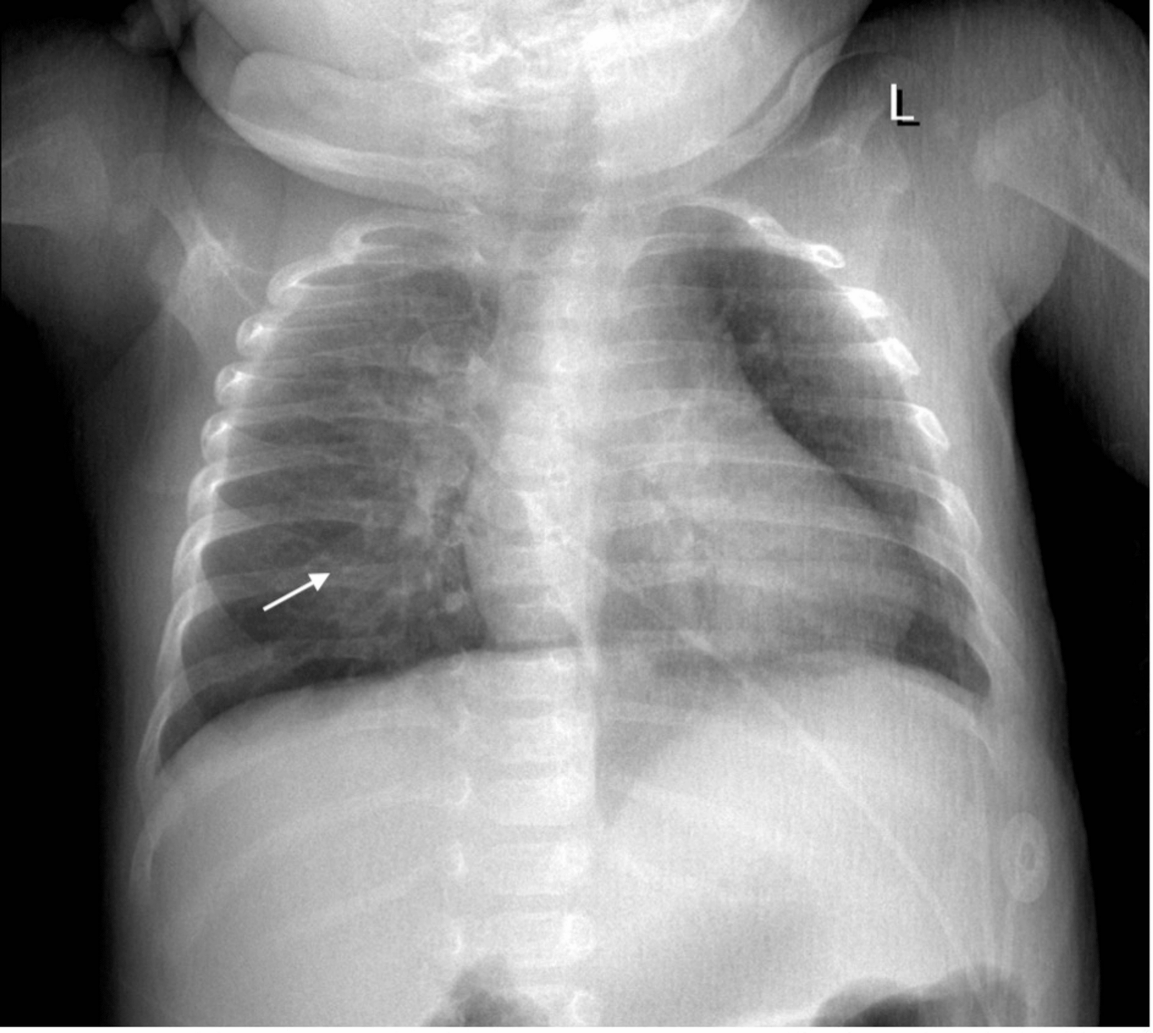
X-ray of the chest showing bilateral patchy airspace opacities as indicated by the white arrow

Three days prior to admission, the patient developed rhinorrhea, nasal congestion, decreased oral intake, and decreased urinary output. On physical examination, the patient had bilateral rhonchi with faint wheezing. She also experienced episodes of perioral cyanosis with associated oxygen desaturations. Chest X-ray showed bilateral patchy airspace opacities concerning for pneumonia (Figure 2).

A complete blood count (CBC) and complete metabolic panel (CMP) were taken on admission (Tables 1-2). A respiratory virus panel was also ordered on admission (Table 3).

**Table 1:** Initial complete blood count (CBC) Abnormal values are in bold.

Lab	Initial value	Normal range
White blood cell count (cells/mcl)	9.38 x 10^9	4.5-10.5 x 10^9
Red blood cell count (cells/mcl)	3.71 x 10^12	4.4 - 6.15 x 10^12
Hemoglobin (gm/dL)	10.1	14.0-18.0
Hematocrit (%)	31.4	40.0-54.0
Mean corpuscular volume (fL)	84.6	81.0-96.0
Mean corpuscular hemoglobin (pg)	27.2	27.0-34.0
Platelet count (platelets/mcL)	229	150-450 x 10^9

**Table 2:** Initial complete metabolic panel (CMP) Abnormal values are in bold.

Lab	Initial value	Normal range
Glucose (mg/dL)	64	74-106
Sodium (mmol/L)	139	135-148
Potassium (mmol/L)	3.8	3.5-5.2
Chloride (mmol/L)	109	95-110
Bicarbonate (mEq/L)	24	21-32
BUN (mg/dL)	6	7-18
Creatinine (mg/dL)	0.3	0.7-1.3

**Table 3:** Respiratory viral panel on admission Abnormal values are in bold.

Virus	Result
Adenovirus	Not Detected
Corona SARS-CoV-2 PCR	Not Detected
Influenza A	Not Detected
Influenza B	Not Detected
Respiratory syncytial virus	Not Detected
Rhinovirus/Enterovirus	Not Detected
Bordetella pertussis	Not Detected
Chlamydophila pneumoniae	Not Detected
Mycoplasma pneumonia	Not Detected
Parainfluenza virus 1	Not Detected
Parainfluenza virus 2	Not Detected
Parainfluenza virus 3	Not Detected
Parainfluenza virus 4	Not Detected

A repeat video swallow study completed during her admission stay was positive for aspiration of liquids of various consistencies and solids. The patient was placed NPO following the study due to an increased risk of silent aspiration. A trial of nasoduodenal (ND) tube feeds was initiated in anticipation of a G-tube placement and Nissen fundoplication. After five days of successful feeds via the ND tube, the G tube was placed, and fundoplication was completed.

## Discussion

Joubert syndrome was first described in 1969 in four siblings with agenesis of the cerebellar vermis that exhibited episodic rapid breathing, abnormal eye movements, ataxia, and mental retardation [6]. Years later, the classic midbrain-hindbrain malformation termed the “molar tooth sign” was detected in Joubert syndrome [5]. This malformation found on radiological imaging is an essential aspect in the diagnosis of Joubert syndrome. The term “Joubert Syndrome and Related Disorders (JSRD) was then used to group all conditions sharing the molar tooth sign [1]. Since 2014, 23 genes have been shown to cause JSRD, with most of these finesse-encoding proteins involved in cilia function or assembly [4].

Joubert syndrome should be considered in infants presenting with hypotonia, abnormal eye movements, irregular respiratory patterns, and developmental delay. In these children, a brain MRI is used to confirm the diagnosis. Features often necessary for diagnosis include cranial MRI findings of midbrain-hindbrain malformation known as the molar tooth sign, hypotonia in infancy, irregular breathing patterns in infancy, and abnormal eye movements [1]. Our case fulfilled the aforementioned criteria.

The clinical manifestations of Joubert syndrome usually begin in the neonatal period; however, the correct diagnosis is typically not made for months or years after birth [7]. In nearly all JSRD patients, early hypotonia can be observed and can be recognized in either the neonatal period or infancy [7]. Hypotonia is a nonspecific finding that can be associated with many other pediatric disorders, therefore other unique features that would suggest JSRD include an irregular breathing pattern and abnormal eye movements. The classic respiratory abnormalities found in Joubert syndrome consist of short alternating episodes of apnea and hyperpnea or episodic hyperpnea alone [2]. Abnormal respirations are typically seen in the neonatal period and improve with age. On admission, our patient had episodic oxygen desaturations with perioral cyanosis. The mother stated her child typically had five to six episodes of cyanosis per day regardless of sitting or supine positioning.

It is unclear if JS characteristics can continue to worsen over time. However, it is thought to be progressive, thus explaining the infants' gradual feeding difficulties after being discharged from the NICU such as the increased frequency and intensity of choking episodes and milk regurgitation through the nostrils. On her initial swallow study completed after birth, the patient had dysphagia with no aspiration. On admission, the swallow study was repeated and the patient was deemed at risk for chronic aspiration due to dysphagia. This finding is likely due to the cerebellum playing a role in motor control of chewing and swallowing; damage to the cerebellum is associated with dysphagia [8]. Given the lack of cerebellar matter associated with the syndrome, it is a plausible explanation for the silent aspiration with delayed, weak, non-productive cough response and difficulty with airway protection seen in this patient.

The prognosis of JS relates to the extent of different organ system involvement and the severity of breathing dysregulation. After the diagnosis of Joubert syndrome has been made, the involvement of other organ systems should be investigated. In JS, death can typically be due to respiratory failure, for which close monitoring for feeding and respiratory problems should be a priority when managing patients with Joubert syndrome [9].

Our case highlights the importance of diagnosing Joubert syndrome early and investigating the involvement of other organ systems such as the retina, kidney, and liver [10]. The prognosis largely depends on the extent of the organ system involvement. We utilized a multidisciplinary approach, including the services of gastroenterology, speech pathology, pulmonology, and neurology, to ensure comprehensive care and management for the patient. By integrating various specialties, we aimed to optimize therapeutic strategies and improve functional outcomes.

## Conclusions

This case report shows the importance of early diagnosis and a team-based approach to managing JS. The patient’s initial presentation included hypotonia, distinctive facial features, respiratory distress, and dysphagia. The diagnosis was confirmed by the "molar tooth sign" on MRI. However, the non-specific clinical symptoms can lead to delays in diagnosis. As seen in this case, JS is a progressive syndrome. Our patient presented with mild dysphagia that quickly progressed to aspiration and respiratory distress. Prompt diagnosis allows for timely interventions improving both quality of life and patient outcomes. Our team of specialists in gastroenterology, speech pathology, pulmonology, and neurology was crucial in managing this case. This team effort addressed the immediate clinical issues and set the stage to meet the patient's needs in the long term.
